# Chronic, Unipolar, Treatment-Resistant Mania: A Case Report and Literature Review

**DOI:** 10.1192/bjo.2022.363

**Published:** 2022-06-20

**Authors:** Yasir Mateen, John Lally, Sumira Qambar Bokhari

**Affiliations:** 1 College of Psychiatrists of Ireland, Dublin, Ireland; 2 College of Physicians and Surgeons Pakistan, Lahore, Pakistan; 3 Mater Misericordiae University Hospital, Dublin, Ireland; 4 Services Institute of Medical Sciences, Lahore, Pakistan

## Abstract

**Aims:**

Chronic mania is variably defined but classically recognized as the presence of manic symptoms for more than 2 years without remission. The reported incidence ranges between 6–15% among all patients with bipolar disorders. Although it has been described in psychiatry literature for a long time, it has not yet found a place in current nosological systems

**Methods:**

We present a 32-year-old single and unemployed man who is supported by his family and living with a sudden-onset, continuous illness of 12 years’ duration characterized by a resistant and markedly euphoric and expansive mood with grandiose delusions. Other features such as distractibility, pressured speech, racing thoughts and psychomotor disturbance remain significant but vary and are more responsive to medical interventions. Psychotic symptoms are largely confined to mood-congruent delusions, grandiose and religious, and are reported to have followed the mood disturbance from early on. There is no history of substance use, past psychiatric or medical illness, or head trauma and no evidence of a neurological cause on workup. This gentleman has been treated with a range of mood stabilizers and antipsychotics and two courses of ECT over the years. In the recent years, he has been on a combination of Clozapine, Valproate, and Pregabalin with relatively favorable but inadequate response and limited functional improvement.

**Results:**

Chronic mania lasting for 12 years, in the absence of an organic cause, despite the use of a wide gamut of modern psychotropics, alone and in combination with ECT, and with adequate compliance is an exceptionally rare entity. It poses manifold challenges both in terms of diagnostic considerations and therapeutic approaches. The overlap of symptoms of mania, schizophrenia, and schizoaffective disorders along with chronicity adds a particular layer of complexity. The hallmark of chronic mania is euphoric and expansive mood along with grandiose delusions and the presentation is relatively less centered on sleep disturbance, hypersexuality, and psychomotor agitation as compared to an acute manic episode. It is distinguished from schizophrenia spectrum disorders as it lacks flat or inappropriate affect, incongruent delusions and disorganized thought. Course of illness, prior mood episodes and family history are important in differentiating from a schizoaffective pattern of disease.

**Conclusion:**

Unremitting mania of this duration is unique in its psychiatric morbidity and devastating in its impact on the individual in terms of psychosocial functioning, quality of life, physical health and safety. It also brings unprecedented stress on the family and other support systems.

